# Editorial: Law and neuroscience: justice as a challenge for neurorights, neurolaw, and forensic psychology, volume II

**DOI:** 10.3389/fpsyg.2026.1949734

**Published:** 2026-08-20

**Authors:** Eric García-López, José M. Muñoz, Karen Herrera-Ferrá, Cristina Nombela Otero, Eduardo Demetrio Crespo

**Affiliations:** 1 Museo Iberoamericano de Neurociencia y Derecho, MIND, Mexico City, Mexico; 2 International Neurotechnology Governance Observatory (INEGOV), International Center for Neuroscience and Ethics (CINET), Tatiana Foundation, Madrid, Spain; 3 Mexican Association of Neuroethics, Mexico City, Mexico; 4 Biological and Health Psychology Department, School of Psychology, Universidad Autónoma de Madrid, Madrid, Spain; 5 Faculty of Legal and Social Sciences, University of Castilla-La Mancha, Toledo, Spain

**Keywords:** forensic psychology, neuroethics, neurolaw, neurorights, neurotechnology

The relationship between Law and Neuroscience has matured beyond its initial fascination with brain images, free will experiments, and the possibility of reading the mind. Today, the field faces a more ambitious challenge: understanding how scientific advances can contribute to a more accurate, humane, and future-oriented conception of justice without reducing persons to their neural substrates. The contributions gathered in this second volume reflect that evolution. Collectively, they move the conversation from *whether* Neuroscience matters for Law to *how* Neuroscience, Psychology, Technology, and Ethics should reshape legal institutions in an era increasingly defined by neurotechnologies and artificial intelligence.

Across diverse topics and methodologies, the contributions collectively challenge overly simplified accounts of the relationship between brain, behavior, and responsibility. Several contributions invite us to reconsider the idea that human behavior, responsibility, or identity can be adequately be explained by the brain in isolation. Guevara and Muñoz provide perhaps the clearest expression of this position. By examining memory modification technologies and their implications for mental integrity and personal identity, they argue that memories are not merely neural traces but embodied and socially embedded phenomena. Their critique of neuroessentialism resonates throughout the volume. Rather than treating the brain as the sole locus of personhood, they invite us to understand Neurorights through a bio-psycho-social lens.

Interestingly, this challenge to reductionism reappears from a very different direction in the work of Mishra et al. Through the concept of *legalomics*, they redirect attention from the brain to the broader biological systems that influence cognition and behavior, particularly the microbiome and the gut–brain axis. Their analysis suggests that future legal and forensic psychology may need to account for a far richer network of biological influences than previously imagined. This perspective is developed further by Logan et al., who propose the *legalome* as an integrative framework that incorporates Neuroscience, Genetics, Epigenetics, Microbiomics, and environmental influences into a unified understanding of criminal behavior and vulnerability. Together, these two articles expand the horizons of neurolaw, moving it beyond the brain and toward a genuinely systemic conception of the human person.

If these contributions broaden our understanding of behavior, other articles ask what happens when technology reaches directly into the spaces where thoughts, identities, and decisions are formed. Popa Tache and Sararu address the challenge of protecting cognitive autonomy in digital environments increasingly shaped by algorithmic influence. Their proposal of “Authenticity-by-Design” moves the Neurorights debate from abstract principles to practical institutional design, suggesting that autonomy should be protected not only by law but also by the architecture of technological systems themselves.

Martín y Ladera et al. continue this discussion through their notion of *digital defencelessness*. While legal debates on privacy have traditionally focused on data Research Topic, they highlight a deeper vulnerability: the tendency of users to establish apparently intimate relationships with generative AI systems that neither understand nor care about them. Their analysis extends Neurorights from laboratories and neurotechnologies into everyday digital life, demonstrating that the protection of mental privacy has become an urgent challenge for ordinary citizens, not only for scholars engaged in debates about emerging neurotechnologies.

The psychological consequences of digital environments are further explored by Seceleanu et al. Although not explicitly framed within Neurorights discourse, their findings on social media addiction, authenticity, affect, and self-image contribute an important psychological dimension to the volume. Their work suggests that contemporary threats to autonomy and wellbeing are often mediated not through direct coercion but through subtle processes involving identity, emotional engagement, and self-perception. In doing so, they provide an empirical complement to the normative concerns raised by other contributions.

Questions of vulnerability and protection occupy a central place in several articles. García-López et al. introduce the concept of *neurovictimology*, shifting attention toward individuals who may become vulnerable not only through crime and trauma but also through the use of neurotechnologies themselves. Their contribution is particularly significant because it broadens a field that has often focused on offenders, responsibility, and punishment. By placing victims at the center of the discussion, they remind us that technological innovation may generate new forms of harm requiring novel forms of protection.

This concern with vulnerability also appears in the work of Haveman et al. Through a dialogue involving clinicians, ethicists, legal scholars, researchers, and experts by experience, they explore the promises and perils of neuroprediction and neuromodulation in forensic care. Their findings reveal cautious optimism: participants recognized the potential benefits of neuro-informed risk assessment and rehabilitation while insisting on the importance of transparency, informed consent, contextual interpretation, and respect for human dignity. In many respects, their article represents the practical counterpart to the more conceptual discussions found elsewhere in the volume, illustrating what responsible translation from laboratory to practice might look like.

The forensic domain receives further attention through contributions addressing cognition, development, and emotional functioning. Meddeb et al. highlight a surprisingly underexplored issue: the lack of conceptual clarity surrounding emotion regulation in forensic populations. Their systematic review demonstrates that before neuroscience can transform forensic practice, fundamental psychological constructs must be measured consistently and rigorously. Similarly, Ioannidis et al. show that understanding offending behavior among individuals with severe mental disorders requires attention to the complex interplay between intelligence and social cognition. Their findings support a more individualized approach to forensic assessment and rehabilitation, moving beyond simplistic diagnostic categories.

Developmental considerations are brought to the forefront by Ramírez-Rivera and Martínez-González, whose analysis of juvenile justice in Puerto Rico illustrates how neuroscience can enrich rather than replace legal reasoning. Their concept of *neurodevelopmental justice* offers a nuanced middle ground between punitive and deterministic approaches, recognizing both adolescents' developmental limitations and their capacity for growth. The article reflects a broader message running throughout the volume: neuroscience is most valuable when it helps refine legal judgment, not when it seeks to substitute it.

This message is perhaps most explicitly articulated by Schleim. Revisiting the enduring debate on free will, he argues that criminal law and everyday moral practice do not depend on resolving metaphysical questions about determinism. What matters are capacities for insight, deliberation, and conscious control. By redirecting attention from abstract philosophical disputes to practical questions of agency and responsibility, Schleim provides a fitting conceptual anchor for the entire Research Topic.

Taken together, the contributions gathered in this volume reveal a field in transition. Neuroscience is no longer presented as a revolutionary force destined to overturn law, nor is it dismissed as irrelevant to legal practice. Instead, these articles demonstrate the emergence of a more mature dialogue in which Neuroscience, Psychology, Technology, and Law are understood as complementary sources of knowledge about human behavior and human dignity.

What ultimately unites the contributions is their shared recognition that justice in the 21st century must confront new realities: minds shaped by digital environments, identities vulnerable to technological intervention, behaviors influenced by biological systems extending beyond the brain, and legal institutions challenged to incorporate scientific knowledge without sacrificing fundamental rights. In exploring these questions, this volume advances not only the fields of Neurorights, Neurolaw, and Forensic Psychology, but also a broader conversation about what it means to protect human agency, autonomy, and dignity in an increasingly neurotechnological world.

